# Mr. James Boyle on Indian Cholera

**Published:** 1821-06-01

**Authors:** 


*A Treatise on the Epidemic Cholera of India.* By James Boyle, Surgeon of His Majesty's Ship Minden, &c. &c. In octavo, pp. 75. London, 1821.

The ravages of the plague, at least in modern times, have produced nothing like the destruction of this Epidemic Cholera; and unfortunately it is not the epidemic of a season, but that of a series of years, of which the termination is not yet in view. Of its etiology we know nothing—or next to nothing; its pathology admits of doubt; and its treatment is but too often unsuccessful. Under these circumstances, we must be contented with such gleams of light as observers have been able to throw upon the nature and management of this formidable disease.

The author of the little volume before us is a meritorious young naval surgeon, who, whilst serving on board His Majesty's Ship Minden, with the Commander in Chief, in the East Indies, had to treat many cases of the Epidemic Cholera, and has here offered the results of his observations and reflections to his brethren at large. We have lately devoted so much space to the consideration of this wide-spreading disease, that we must be very limited in our analytical notice of the tract under review.

Mr. Boyle thinks that more depends on combating casual symptoms as they occur, in this disease, than on any systematic mode of treatment. Our author makes many judicious observations on the remote causes of Indian Cholera, especially drunkenness, marsh effluvia, and constipation of the bowels, sustaining his remarks by appropriate cases. His *post mortem* investigations do not materially differ from those which we have given an ample account of in this Journal. The following fact has been very generally confirmed by all accurate observers.

> "It sometimes happens, that patients despaired of, have a critical evacuation of viscid bile, exactly resembling that which has been noticed in the gall bladder of those who had died of the disease, with obstruction of the biliary ducts. When this circumstance takes place, the patient invariably recovers; and I have known it to occur three or four times, in cases where the pulse had been almost imperceptible for twenty-four hours." P. 51.

Bleeding, Mr. Boyle thinks, is a remedy applicable only to the milder cases of this destructive cholera. Before reaction he considers the remedy useless, if not pernicious;—after that process has taken place, he imagines the original disease no longer exists.

> "The great extent to which mercury has been carried of late years by the first practitioners, and it having now gained the ascendency of popular opinion over vulgar prejudice and inconsistent objections, has a just claim for consideration as a remedial measure in the management of this complaint. The universally acknowledged circumstance of the absence of bile in bad cases of Cholera, and the more than idea that mercury acts specifically on the liver and biliary system, are also strong incentives in favour of its use. Fatal cases, however, but two frequently occur where calomel is rejected as fast as it is given; or if retained, is found, on examination after death, to have insinuated itself between the rugæ of the stomach, perfect in appearance, and without having had any effect whatever. Mercury, therefore, ought not to be depended on in this disease as long as nausea and sickness of stomach prevail, and to restrain which, by sedatives, appears almost impossible in severe cases, till all sensibility be destroyed, when it matters not what is given." 56.

The warm bath, he observes, in dangerous cases is always advisable; "but measures of greater importance should be actively employed during its preparation." Mr. Boyle never observed any benefit from the bath, unless it was so graduated as to make the patient complain of its heat.

"When the bath is of such temperature as to be only agreeable to the person, the remedy appears altogether inert; but when, from heat and the patient's sensibility, remonstrance and even force is necessary to keep him in it, the result is usually favourable. 57.

Our author remarks, at page 58, that "the grand, primary, and most essential object in the treatment of cholera morbus is, *to restore the balance of the circulation* as quickly as possible." This is, as he observes, a general opinion; but unfortunately the restoration is not so easily effected.

"The constant nausea and irritation of stomach, which is observable in the early stages of this complaint, without full or violent vomiting, simply spouting up, as it were, any thing swallowed; the obstruction of the biliary ducts observed in dissection, and a general want of success in practice; induced me to embrace ideas perfectly new on the subject. The obstruction of the biliary ducts I looked on as a source of irritation to the nervous system generally, and the nausea and sickness of stomach as an effort of nature to free herself of an unaccustomed evil.

"In accounting for the causes of this disease, it has been observed, and with great justice, that when, from the exertions to vomit, bile makes its appearance, a favourable prognosis may be formed. Now, if the appearance of bile be a salutary one, (and it certainly is,) why not favour the progress of its formation, instead of obstructing its passage by the administration of sedatives? We know of nothing which will increase the secretion of bile so quickly or so effectually as the act of vomiting; we also know the sympathy which subsits between the liver and stomach, and that derangement of either organ will more or less affect both. It is evident, then, that the gastric derangement peculiar to this disease, is not only indicative of the existence of lurking mischief, but directly points to the treatment. Further, of all the cases of which I have seen or heard, there was not one fatal termination after bile had, in any way, or by any means, made its appearance." 61.

Reflecting on these circumstances, our author was led to try *emetics* as the most likely measure to answer the various purposes of clearing the stomach, removing obstructions of the biliary ducts, and exciting a new action in the vascular system. We shall state the first case that presents itself as a specimen of all.

"*Minden, at Trincomalee, Feb. 28th,* 1821.

"James Leister (S) complained at two P. M. of head-ache and pain of bowels, with thirst, and tottering of the limbs. The pulse was small and feeble; the skin was particularly cold; and the countenance greatly dejected. Cramps of the toes, fingers, and abdominal muscles, soon succeeded this state; followed by nausea, sickness of stomach, and a peculiar shrivelled appearance of the integuments of the fingers. The pulse at the wrist was scarcely to be felt; the extremities were apparently lifeless; eyes dull and fixed, exhibiting a shining, glassy appearance; with a collapsed, dark-coloured countenance, and constant attempts to vomit. Gave him antim. tart. in repeated small doses, for the purpose of causing full vomiting. This not having the desired effect, five grains were given at once: still, however, the efforts to vomit were faint and ineffectual. Pulv. ipecac. ℈j. statim. This, followed up by copious draughts of warm water, caused violent retching and vomiting. During this time, the feet were placed in pediluvium; a hot bath was ordered to be in readiness; and the patient having been placed in bed, was allowed to drink freely of arrack punch with spt. lavend. comp. An ounce of mercurial ointment was rubbed over the region of the liver in one hour; at the expiration of which time, reaction had tolerably established itself. The voice was yet low and indistinct; and he had partial, cold, clammy perspirations. He was now placed in the hot bath, with the intention of keeping up the action of the heart, and influencing the operation of the mercury. A purgative enema was thrown up, which was followed by a copious, thin clay-coloured evacuation. After this, what was passed had more the appearance of congee water* than any thing else to which I can compare it.

"At six P. M. pulse still feeble, but distinct; voice returning; Complains only of debility and pain of bowels. Hab. statim subm. hyd. ℈j. At ten, had several thin dejections, but without a particle of bile, and still complains of pain of bowels. Applicet. emp. lyttæ ad region. abdomin. et repet. subm. hyd.

"29th. Complains of great debility. Pulse still small and feeble; skin moderately warm, but dry with thirst. Had a purgative at four A. M. which operated. Still no bile. Repet. subm. hyd.

"1st. of March. Still extremely languid, although free from pain. No bile in his evacuations; nor is the mouth affected. Pil. alter. ter in die.

"2nd. Mouth slightly sore; evacuations natural. Omit the pill, et habeat inf. quassiæ.

"Was discharged to duty on the eighth following." P. 64.

---

* A name given to rice-water in India.

Sincerely do we hope that the measure suggested and adopted by our author may be found serviceable in the hands of others also. If such should be the case, Mr. Boyle will be entitled to the esteem of his professional brethren in the Eastern world, to whom we recommend this sensible and unostentatious little treatise.

